# Thrombospondin-4 drives lymphangiogenesis through cooperation with VEGF-C in human bladder cancer

**DOI:** 10.7150/ijms.122895

**Published:** 2026-01-01

**Authors:** Thomas I-Sheng Hwang, Pei-Wen Peng, Mau-Shin Chi, Kuang-Yu Chou, Te-Fu Tsai, Chao-Yen Ho, An-Chen Chang

**Affiliations:** 1 Division of Urology, Department of Surgery, Shin Kong Wu Ho-Su Memorial Hospital, Taipei 111045, Taiwan, R.O.C.; 2 Division of Urology, School of Medicine, Fu Jen Catholic University, New Taipei 242062, Taiwan, R.O.C.; 3 Department of Urology, Taipei Medical University, Taipei 110301, Taiwan, R.O.C.; 4 School of Dental Technology, Taipei Medical University, Taipei 110301, Taiwan, R.O.C.; 5 Department of Radiation Therapy and Oncology, Shin Kong Wu Ho-Su Memorial Hospital, Taipei 111045, Taiwan, R.O.C.; 6 Institute of Veterinary Clinical Science, School of Veterinary Medicine, National Taiwan University, Taipei 106319, Taiwan, R.O.C.; 7 Institute of Traditional Medicine, School of Medicine, National Yang Ming Chiao Tung University, Taipei 112304, Taiwan, R.O.C.; 8 School of Oral Hygiene, College of Oral Medicine, Taipei Medical University, Taipei 110301, Taiwan, R.O.C.; 9 Stem Cell Research Center, College of Oral Medicine, Taipei Medical University, 110301, Taipei, Taiwan, R.O.C.; 10 Translational Medicine Center, Research Department, Shin Kong Wu Ho-Su Memorial Hospital, Taipei 111045, Taiwan, R.O.C.

**Keywords:** bladder cancer, thrombospondin-4, lymphangiogenesis

## Abstract

**Introduction:** Bladder cancer (BLCA) is the second most common malignancy of the male urinary tract, with frequent metastasis to pelvic lymph nodes and, in advanced stages, to distant organs such as the lungs, liver, and bones. Thrombospondin-4 (TSP4), an extracellular matrix protein, has been implicated in cell adhesion, migration, proliferation, and cytoskeletal regulation. However, its role in lymphatic metastasis remains poorly understood.

**Methods:** TSP4 mRNA and protein levels were assessed by RT-qPCR and Western blot analyses. Lymphangiogenesis and lymphatic endothelial cell (LEC) migration were respectively evaluated using tube-formation and transwell assays. The Cancer Genome Atlas (TCGA)-BLCA datasets were analyzed to compare expressions of TSP family members across lymph node metastasis stages. A popliteal lymph node metastasis model was employed to isolate lymph node-tropic BLCA cell lines.

**Results:** TSP4 expression was positively associated with lymph node metastasis stages in BLCA tissues. In vitro, conditioned medium (CM) from 5637 and T24 cells promoted LEC recruitment and tube formation, while a TSP4-neutralizing antibody impaired LEC migration without affecting tube formation. Mechanistically, TSP4 enhanced LEC migration by upregulating intercellular cell adhesion molecule (ICAM)-1 and activating the integrin αvβ3/focal adhesion kinase (FAK)/extracellular signal-regulated kinase (ERK) pathway. Notably, TSP4 also induced vascular endothelial growth factor C (VEGF-C) expression in BLCA cells. Both TSP4 and VEGF-C were upregulated in lymph node-tropic BLCA cell lines, with a positive correlation observed in TCGA-BLCA datasets. VEGF-C neutralization abolished CM-induced LEC tube formation, highlighting the role of the TSP4/VEGF-C axis in lymphangiogenesis.

**Conclusions:** This is the first study to demonstrate that BLCA-derived TSP4 cooperates with VEGF-C to promote lymphangiogenesis within the tumor microenvironment. These findings suggest that TSP4 could serve as a potential therapeutic target for preventing lymphatic metastasis in human BLCA.

## Introduction

Bladder cancer (BLCA) is the fourth most common malignancy of the urinary tract and one of the leading causes of cancer-related deaths in men 1. Approximately 70%-75% of BLCA cases are classified as non-muscle-invasive bladder cancer (NMIBC), 20%-25% as muscle-invasive bladder cancer (MIBC), and 5% as metastatic disease, each representing a distinct stage of disease progression. Treatments for patients with high-grade T1 BLCA include transurethral resection of the bladder tumor (TURBT), adjuvant chemotherapy with gemcitabine and cisplatin, and Bacille Calmette-Guérin (BCG) therapy for up to 3 years 2. However, 5-year recurrence and progression rates remain high at 42% and 21%, respectively 3. These rates highlight the critical prognostic significance of lymph node metastasis (LNM) in high-grade T1 BLCA 4. Studies showed that the presence of LNM drastically reduces the 5-year cancer-specific survival rate from 77.6% to 18.6% 5. Nevertheless, the specific molecular mechanisms that trigger lymphatic spread in BLCA remain poorly understood. Therefore, elucidating the mechanisms underlying LNM in BLCA is essential for developing novel therapeutic strategies to prevent malignant progression.

The lymphatic system plays a vital role in immune function, tissue fluid homeostasis, and the absorption of fatty acids in the human body 6. The process of lymphangiogenesis involves the formation of new lymphatic vessels, occurring during embryonic development, wound healing, and various pathological conditions 7. During tumor progression, cancer cells and cells within the tumor microenvironment (TME) produce lymphangiogenic factors—such as vascular endothelial growth factor (VEGF)-C, fibroblast growth factor (FGF)-2, and platelet-derived growth factor (PDGF)-BB—that attract lymphatic endothelial cells (LECs) to infiltrate the tumor tissues, resulting in the formation of new lymphatic vessels within and around the tumor. This ultimately facilitates the metastasis of cancer cells to distant organs via the lymphatic system 8, 9. Numerous cell and animal studies confirmed that VEGF-C can promote distant metastasis of tumors 10, 11. When VEGF-C signaling is blocked by neutralizing antibodies, lymphatic metastasis is suppressed 12, 13. This suggests that VEGF-C offers a potential therapeutic target for tumor progression and metastasis.

The TSP family comprises extracellular calcium-binding proteins, consisting of five matricellular proteins (TSP-1, -2, -3, -4, and -5) 14. TSP proteins interact with various membrane proteins on the cell surface to regulate cell phenotypes and the extracellular matrix (ECM) architecture 15-17. Recently, in the context of tumor progression, expressions of specific TSPs were associated with tumor growth, metastasis, and shortened survival 18, 19. In 2021, our research team published a study on TSP4 in BLCA, demonstrating that TSP4 is highly expressed in BLCA tissues and is significantly associated with poor patient prognoses 20. Those findings suggested that TSP4 may serve as a potential prognostic marker for BLCA. Furthermore, through a series of in vitro experiments, we confirmed that TSP4 promotes matrix metalloproteinase 2 (MMP2) expression via the AKT signaling pathway, thereby enhancing the migratory ability of BLCA cells. Although the role of the TSP protein family in tumor progression has been studied, their specific mechanisms in lymphatic metastasis remain largely unexplored.

In this study, we attempted to elucidate how TSP4 promotes lymphangiogenesis in BLCA by recruiting LECs via the integrin αvβ3/focal adhesion kinase (FAK)/extracellular signal-regulated kinase (ERK)/intercellular adhesion molecule (ICAM)-1 signaling pathway. TSP4 also cooperates with VEGF-C to regulate LEC activity and enhance lymphatic vessel formation. Lymph node-tropic BLCA cells were found to exhibit elevated expressions of both TSP4 and VEGF-C. This is the first study to demonstrate the TSP4/VEGF-C axis as a driver of both LEC recruitment and tube formation. These findings highlight TSP4 as a potential therapeutic target for preventing lymphatic metastasis in human BLCA.

## Materials and Methods

### Cell culture

The 5637 human BLCA cell line was cultured in RPMI-1640 medium (Gibco, Thermo Fisher Scientific). The RT4 and T24 human BLCA cell lines were cultured in McCoy's 5A medium (Gibco, Thermo Fisher Scientific). Human LECs were cultured in EGM-2 MV BulletKit medium (Lonza Bioscience), with culture dishes pre-coated with 1% gelatin (Sigma-Aldrich) for subculturing. All culture media were supplemented with 10% fetal bovine serum (FBS; Gibco), 2 mM GlutaMAX-1, 100 units/mL penicillin, and 100 μg/mL streptomycin (Gibco). All cells were maintained in a humidified incubator at 37 °C with 5% CO_2_.

### Conditioned medium (CM) collection

The RT4, T24, and 5637 BLCA cell lines were seeded at a density of 2 × 10⁶ cells/10 mL in 10-cm dishes and incubated for 24 h. The medium was then replaced with serum-free medium, and after an additional 48 h, CM was collected and designated RT4 CM, T24 CM or 5637 CM.

### Western blot assay

Proteins extracted from BLCA cells were quantified using a BCA Protein Assay Kit (Thermo Fisher Scientific), separated by sodium dodecylsulfate-polyacrylamide gel electrophoresis (SDS-PAGE), and transferred onto polyvinylidene fluoride (PVDF) membranes (Thermo Fisher Scientific). The membrane was then blocked with 5% skim milk at room temperature for 1 h, followed by incubation at 4 °C overnight with anti-TSP4 (1:3000; GTX125869, GeneTex), anti-ICAM-1 (1:3000; GTX100450, GeneTex), and anti-β-actin (1:5000; GTX109630, GeneTex) primary antibodies. After three washes with PBST (PBS and 0.05% Tween 20), the membrane was incubated at room temperature for 1 h with horseradish-peroxidase-conjugated anti-mouse (1:3000; GTX213111-01, GeneTex) and anti-rabbit (1:3000; GTX213110-01, Genetex) secondary antibodies. Protein signals were detected using the ImageQuant LAS 4000 biomolecular imager (GE Healthcare Life Sciences), and protein expression levels were quantified with UN-SCAN-IT gel analytical software (vers. 6.1).

### Real-time reverse-transcription quantitative polymerase chain reaction (RT-qPCR)

Total RNA was purified from cells using the TRIzol reagent (Thermo Fisher Scientific) and quantified with Nanodrop (Thermo Fisher Scientific), using 1 μg of RNA for subsequent analyses. Complementary (c)DNA was synthesized from RNA using the Magic RT Master Mix cDNA Synthesis Kit (Invitrogen, Thermo Fisher Scientific). For the RT-qPCR, sequence-specific primers for human TSP4 (forward, 5′-TGCTGCCAGTCCTGACAGA′; reverse, 5′-GTTTAAGCGTCCCATCACAGTA-3′) or GAPDH (forward, 5′-TGTGGGCATCAATGGATTTGG-3′; reverse, 5′-ACACCATGTATTCCGGGTCAAT-3′), SYBR Green Master Mix (Applied Biosystems, Thermo Fisher Scientific) and 100 ng of cDNA were pre-mixed. The RT-qPCR was performed on a StepOnePlus system in a three-step protocol: initial polymerase activation at 95 °C for 10 min, followed by 40 amplification cycles consisting of denaturation at 95 °C for 15 s and extension at 60 °C for 60 s. Gene expression levels were analyzed based on cycle threshold (CT) values. Expression levels of TSP4 were assessed using the 2-ΔΔCq method to quantify transcript levels, with GAPDH serving as the internal control.

### LEC recruitment assay

LECs were seeded in the upper chamber of a Transwell insert (pore size, 8 µm; Costar), while 300 μL of culture medium containing 1% FBS or recombinant TSP4 protein (30 ng/mL) was added to the lower chamber. After incubation for 24 h at 37 °C, cells were fixed with 3.7% formaldehyde (Sigma-Aldrich/Merck) for 15 min, followed by staining with 0.05% crystal violet (Sigma-Aldrich) for 20 min. The number of migrated LECs was then counted and subjected to a quantitative analysis.

### Tube-formation assay

In total, 100 μL of Corning® Matrigel® Matrix was added to each well of a 48-well plate and incubated at 37 °C for 1 h. LECs were pre-labeled with calcein AM (1 μg/mL) and 4×10^4^ cells were counted and mixed with 100 μL of CM from BLCA cells. The mixture was then seeded into Matrigel-coated wells and incubated for 6 h. Tube formation was imaged, and the total tube length was quantified using ImageJ software.

### Establishment of lymph node-tropic BLCA cell lines

The animal study protocols were approved by the Institutional Animal Care and Use Committee (No. 114SKH010); the animal experiments were performed in compliance with the Guidelines of Animal Experimentation set forth by Shin Kong Wu Ho-Su Memorial Hospital. BLCA cells expressing luciferase (UMUC3-Luc, 1x10⁶ cells) were injected into the footpads of 6-week-old nude mice. After 6 weeks of stable tumor growth, the mice were sacrificed, and tumor tissues that had metastasized to popliteal lymph nodes (PLNs) were harvested for cell culture. Once cells had expanded, a second round of footpad injections was performed. Following another 6 weeks of stable tumor growth, the mice were again sacrificed, and tumor tissues from the PLNs were collected for cell culture. Two BLCA cell lines that had successfully metastasized to PLNs in individual mice were isolated and designated as BLCA-LN1 and BLCA-LN2.

### Bioinformatic analysis of The Cancer Genome Atlas (TCGA)-BLCA dataset

Clinical and gene expression data for BLCA (TCGA-BLCA) were obtained from the Broad GDAC Firehose platform (https://gdac.broadinstitute.org/) to analyze expression profiles of TSP family proteins in relation to LNM in BLCA.

### Statistical analysis

All experimental procedures were performed in at least three independent experiments, each with triplicate samples. Statistical significance between two groups was determined using Student's *t*-test, while comparisons among more than two groups were assessed using a one-way analysis of variance (ANOVA). A *p* value of < 0.05 was considered statistically significant.

## Results

### Positive association between THBS4 overexpression and LNM in BLCA

**Figure 1:**
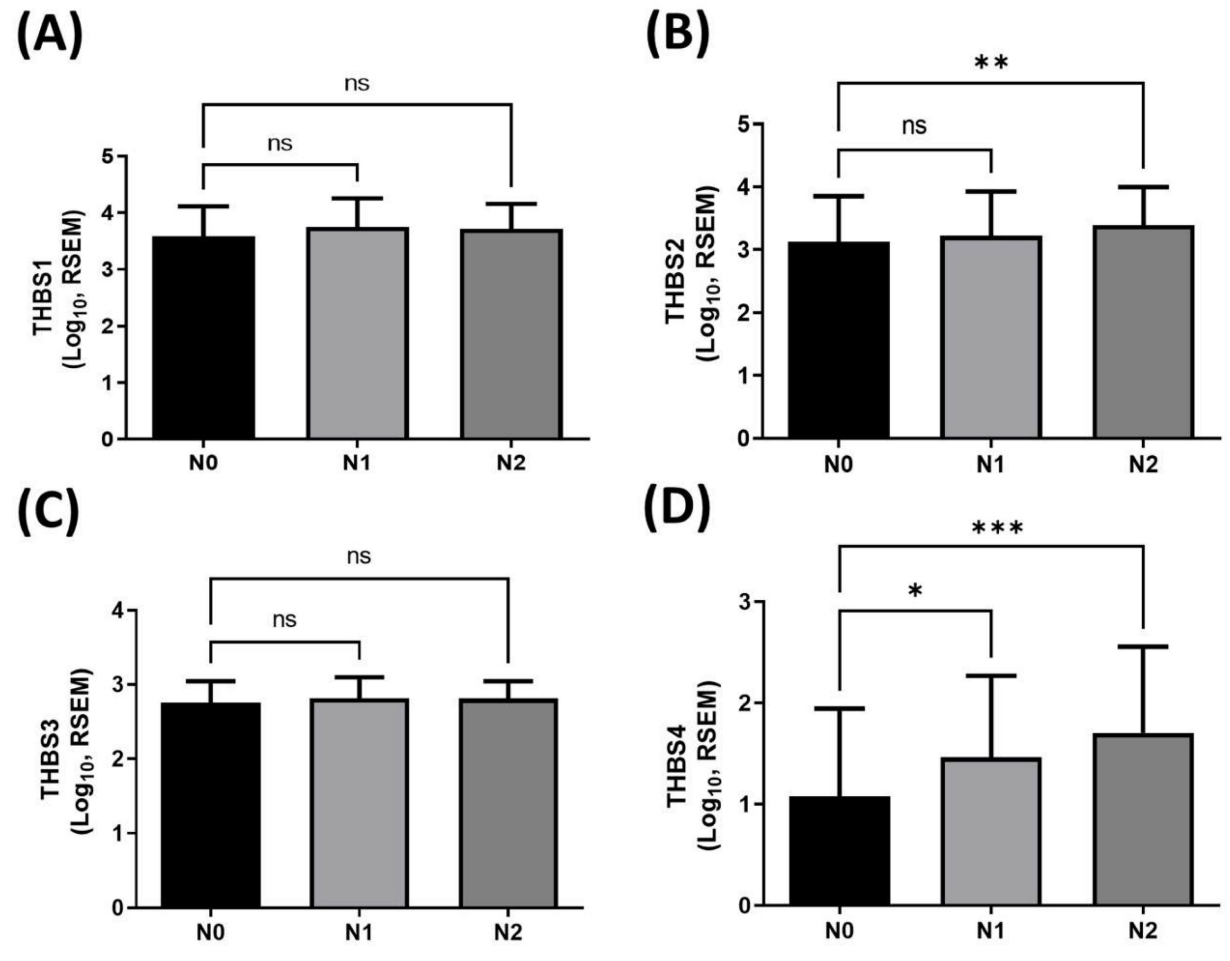
** The clinical importance of THBS genes in lymphatic metastasis in BLCA.** (A-D) Expression levels of THBS1-4 in human BLCA tissues classified into N0 (n = 237), N1 (n = 45), and N2 (n = 75) stages. Data are presented as mean ± SD values. * p < 0.05, ** p < 0.01, and *** *p* < 0.001, compared to the N0 group. (RSEM: RNA-Seq by Expectation-Maximization; N0: no regional lymph node metastasis; N1: metastases in one to three axillary lymph nodes; N2: metastases in four to nine axillary lymph nodes).

To identify which member of the thrombospondin (TSP/*THBS*) gene family may be involved in regulating lymphatic metastasis in human BLCA, we first analyzed expression profiles of *TSP* (*THBS*) genes in tumor tissues using TCGA-BLCA dataset. This dataset includes 237 N0 (no lymph node metastasis), 45 N1, and 75 N2 BLCA cases. RNA sequencing analysis revealed a significant positive correlation between the expression level of TSP4 (*THBS4*) and lymph node metastasis in BLCA (Fig. 1D), whereas no significant differences were observed for TSP1-3 (*THBS1-3*) (Fig. 1A-C). These findings suggest that TSP4 may play a role in regulating lymphatic metastasis in BLCA.

### Cancer-derived TSP4 functions as a lymphangiogenic factor

**Figure 2:**
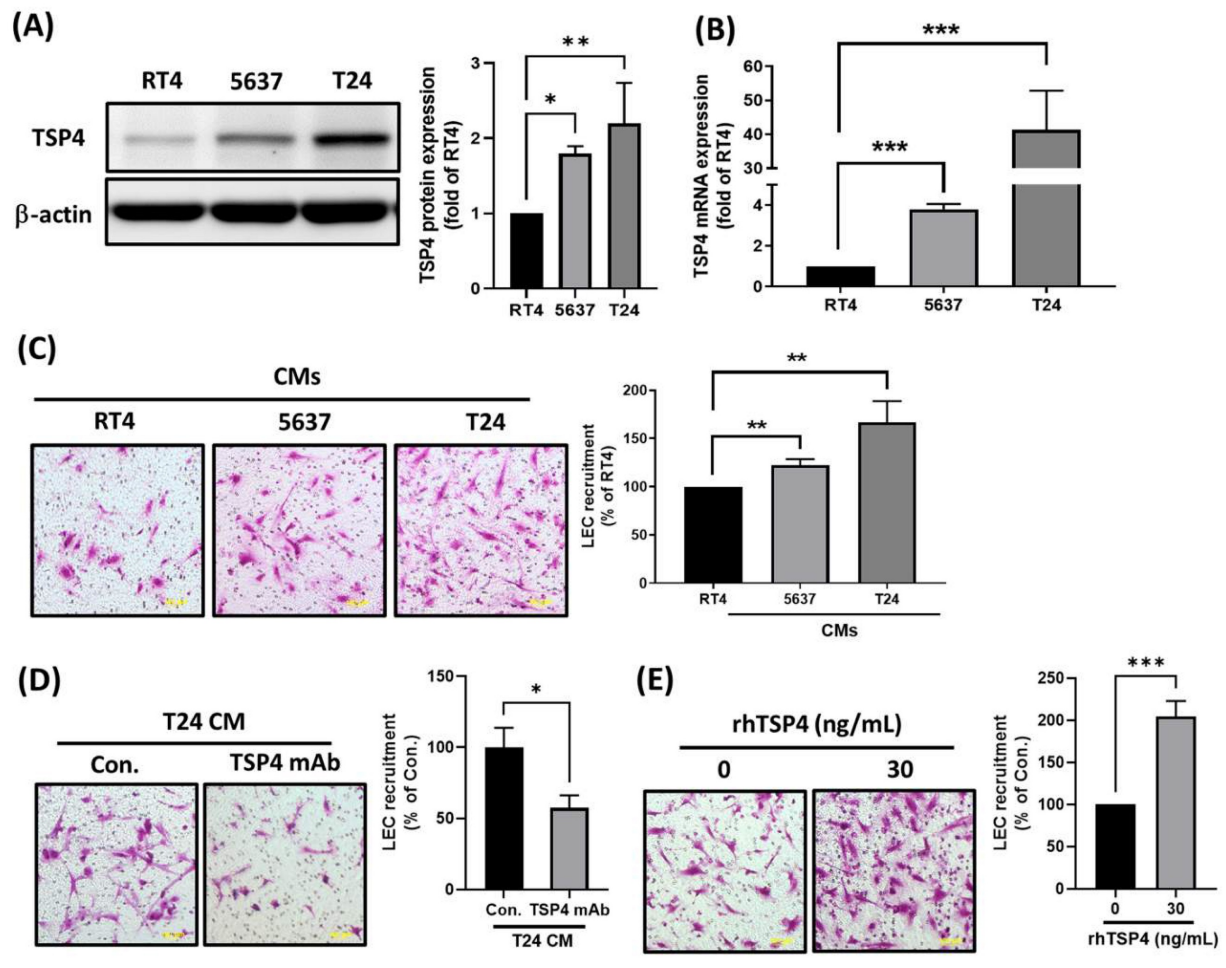
** TSP4 promotes LECs recruitment into the BLCA microenvironment.** (A, B) Protein and mRNA expression levels of TSP4 in BLCA cells were respectively analyzed by Western blotting and a RT-qPCR. (C) CMs from RT4, 5637, or T24 were added to the lower chamber of a Transwell system. LECs were seeded in the upper chamber, and migrated LECs were quantified (scale bars, 100 µm). (D) T24 CMs were pretreated with a TSP4 monoclonal antibody (0.5 μg/mL) for 30 min and then added to the lower chamber. LECs were seeded in the upper chamber, and migrated LECs were quantified (scale bars, 100 µm). (E) LECs were pretreated with or without rhTSP4 (30 ng/mL), and a cell recruitment assay was performed to analyze LEC migration (scale bars, 100 µm). Data are presented as mean ± SD values. * *p* < 0.05, ** *p* < 0.01, and *** *p* < 0.001, compared to the control group.

To preliminarily investigate the mechanism by which TSP4 regulates lymphatic metastasis in BLCA, we performed a series of *in vitro* cellular experiments. First, we examined TSP4 protein and mRNA expressions across BLCA cell lines representing different clinical stages—RT4 (stage I), 5637 (stage II), and T24 (stage III)—using Western blot and RT-qPCR analyses. Results showed that both TSP4 protein and mRNA expressions were positively correlated with the clinical stage of BLCA cells (Fig. 2A, B). To investigate whether cancer-derived TSP4 functions as a lymphangiogenic factor, we collected CMs from the three cancer cell lines and assessed their ability to recruit LECs using a transwell migration assay. Results demonstrated that CM from T24 cells recruited the highest number of LECs, followed by those from 5637 and RT4 cells (Fig. 2C). Moreover, when TSP4 in the T24 CM was blocked using a monoclonal antibody (mAb), the recruitment of LECs was significantly inhibited (Fig. 2D). Furthermore, direct administration of 30 ng/mL recombinant human (rh)TSP4 induced LEC migration (Fig. 2E). Taken together, these results suggested that BLCA cells secrete TSP4 to recruit LECs into the TME, thereby potentially promoting lymphangiogenesis and contributing to lymphatic metastasis.

### Cancer-derived TSP4 induces ICAM-1 expression in LECs

**Figure 3:**
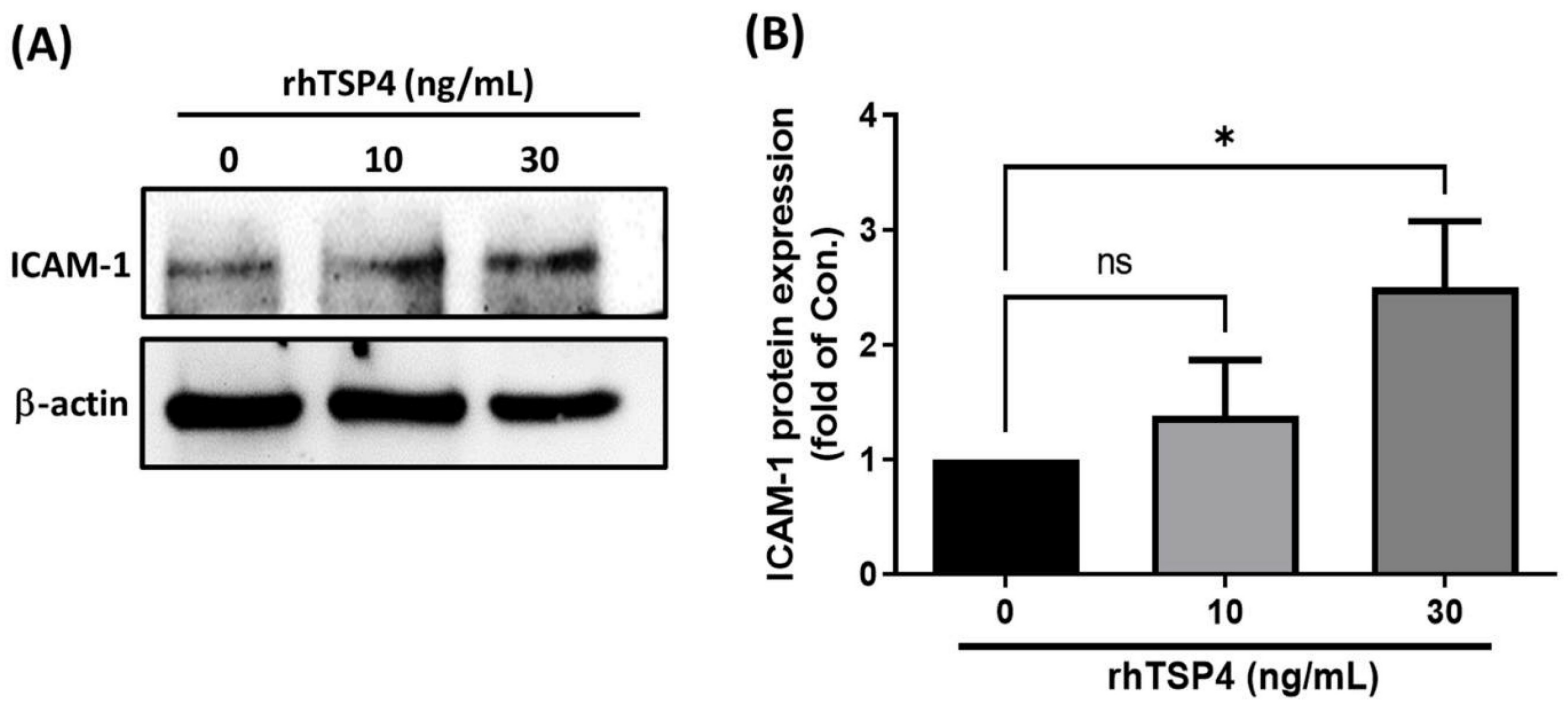
** TSP4 induces ICAM-1 protein expression in human LECs.** (A) LECs were treated with different concentrations of rhTSP4 (0, 10, and 30 ng/mL) for 24 h, followed by analysis of ICAM-1 protein expression levels via Western blotting. (B) Indicated protein expression levels were quantified using UN-SCAN-IT gel 6.1 software. Data are presented as mean ± SD values. * *p* < 0.05, compared to the control group.

A literature review revealed that ICAM-1 plays a crucial role in regulating cell migration in various cell types, including cancer cells, monocytes, and endothelial cells 21-23. In the present study, we therefore determined whether ICAM-1 was a critical mediator of cancer-derived TSP4. LECs were treated with different concentrations of rhTSP4, and results demonstrated a concentration-dependent increase in ICAM-1 protein expression (Fig. 3A, B). These findings suggested that BLCA cell-derived TSP4 may induce ICAM-1 expression in LECs, thereby facilitating their migration into the TME.

### BLCA-derived TSP4 promotes LEC mobility via the ανβ3 integrin

**Figure 4:**
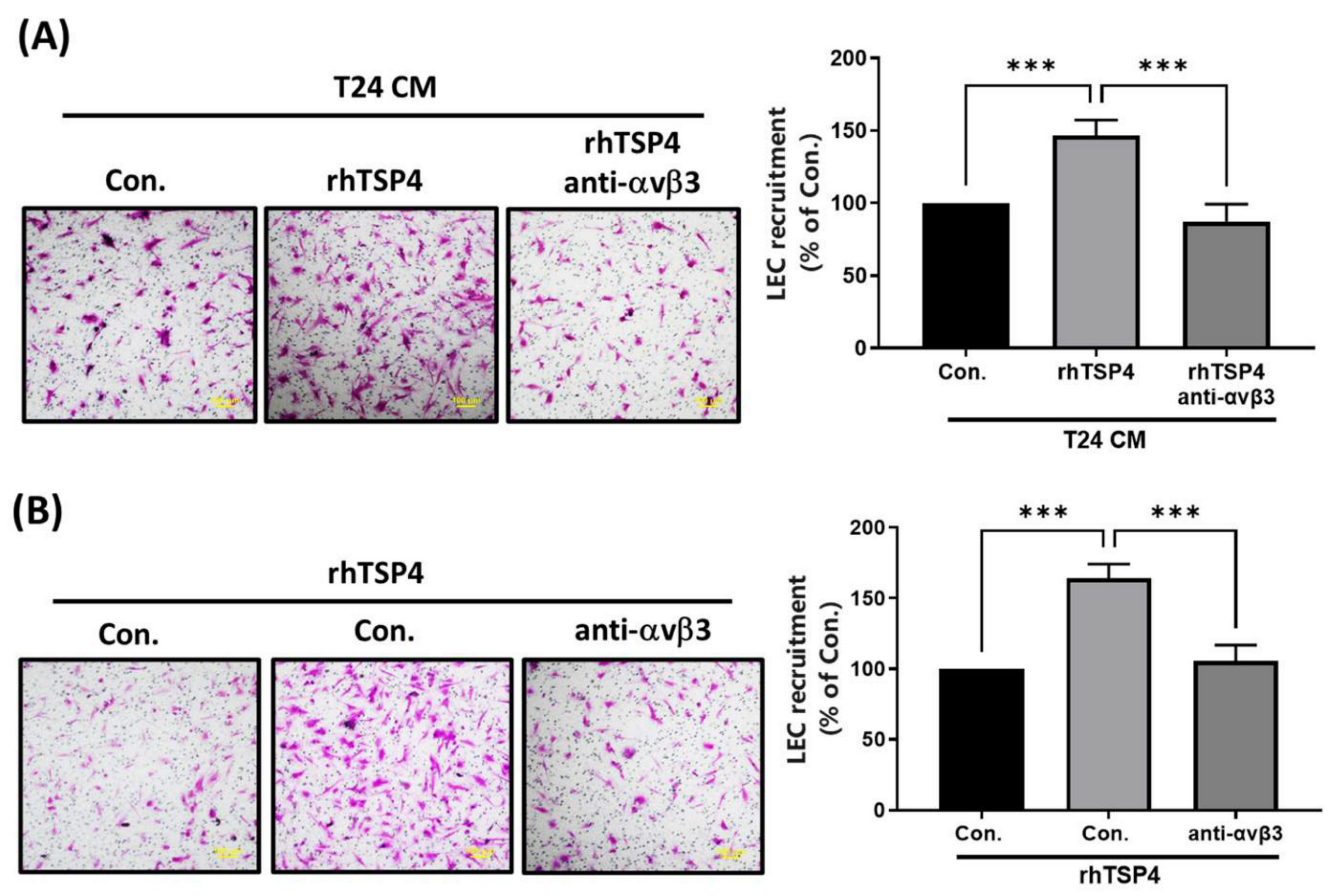
** Integrin αvβ3 is essential in TSP4-relulated LEC motility.** (A) T24 cells were treated with rhTSP4 (30 ng/mL), and the resulting CM were collected as T24 CM. Subsequently, LECs were pretreated with an integrin αvβ3 antibody (0.5 μg/mL) for 30 min, followed by stimulation with T24 CM to assess LEC motility via a recruitment assay (scale bars, 100 µm). (B) LECs were pretreated with an integrin αvβ3 antibody (0.5 μg/mL) for 30 min, followed by treatment with rhTSP4 (30 ng/mL). Cell motility was then assessed using a recruitment assay (scale bars, 100 µm). Data are presented as mean ± SD values. *** *p* < 0.001, compared to the control group.

Integrin αvβ3 was identified as a TSP4-specific receptor that contributes to regulating cell migration and invasion 24. In the present study, we conducted experiments using an mAb against integrin αvβ3 to investigate whether it is involved in TSP4-mediated recruitment of LECs. Results demonstrated that pretreatment with the integrin αvβ3 mAb significantly reduced LEC migration induced by either T24 CM or rhTSP4, as shown in Fig. 4A and 4B. These findings suggested that integrin αvβ3 expressed on LECs plays a critical role in TSP4-mediated recruitment mechanisms.

### TSP4 promotes LEC mobility via activation of the integrin αvβ3/FAK/ERK signaling pathway

**Figure 5:**
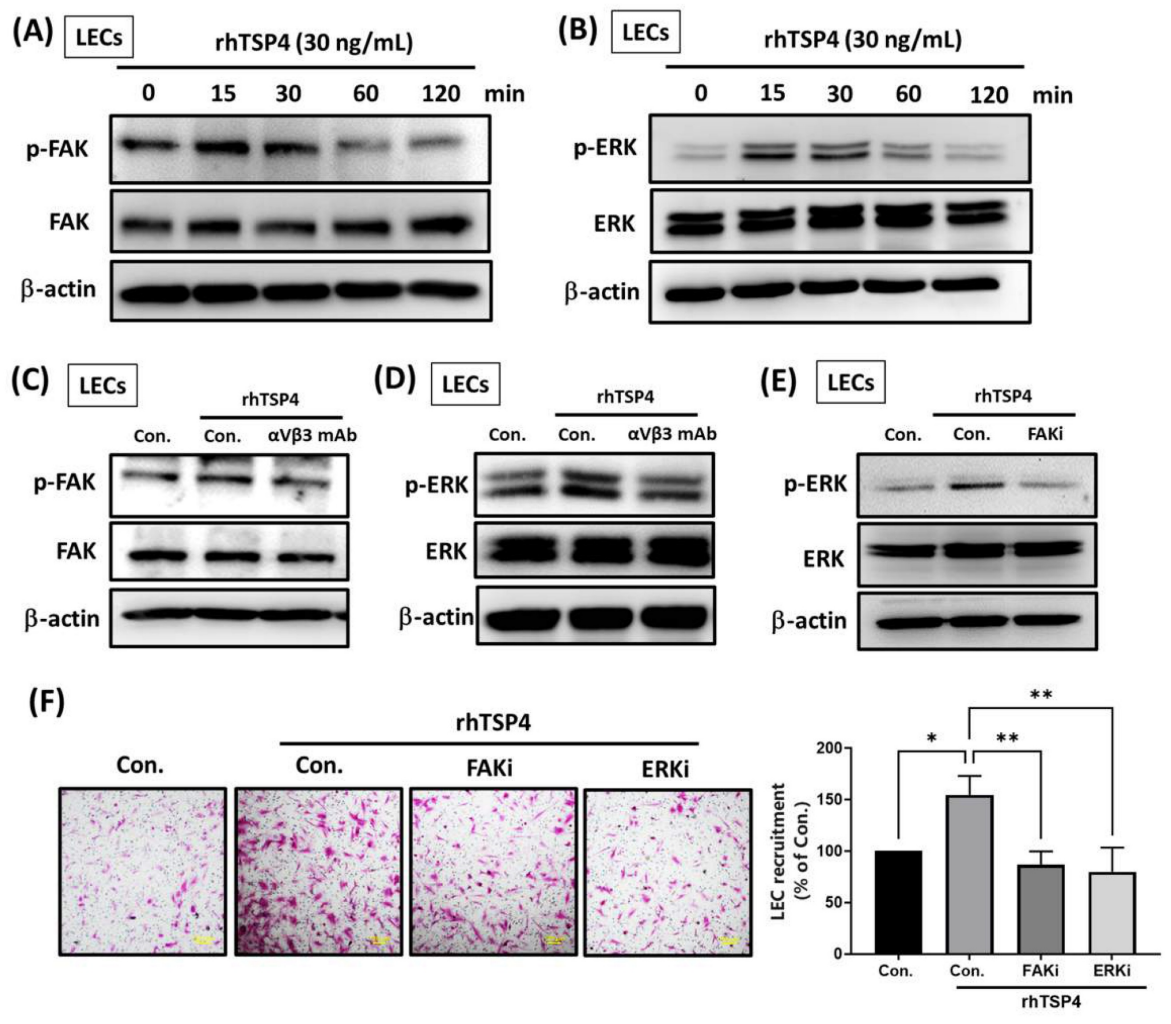
** The integrin αvβ3/FAK/ERK pathway is involved in TSP4-promoted LEC motility.** (A, B) LECs were incubated with rhTSP4 (30 ng/mL) for indicated time points, and expression levels of phosphorylated (p)-FAK and p-ERK were evaluated by Western blotting. (C, D) LECs were pretreated with an integrin αvβ3 antibody (0.5 μg/mL) for 30 min, followed by stimulation with rhTSP4 for 15 min. Expressions of p-FAK and p-ERK were then analyzed by Western blotting. (E) LECs were pretreated with a FAK inhibitor (FAKi, 1 μM) for 30 min, followed by rhTSP4 (30 ng/mL) stimulation for 15 min. p-ERK protein levels were analyzed by Western blotting. (F) LECs were treated with a FAKi (1 μM) and an ERK inhibitor (ERKi, 1 μM), followed by rhTSP4 (30 ng/mL) treatment for 24 h. LEC motility was assessed using a recruitment assay (scale bars, 100 µm). Data are presented as mean ± SD values. * *p* < 0.05, ** *p* < 0.01, compared to the control group.

Intracellular signaling pathways are crucial for cellular biological functions by activating transcription factors and upregulating gene expressions 25. To determine whether intracellular signaling is involved in TSP4-induced LEC mobility, we treated LECs with rhTSP4 at various time points. Results showed that TSP4 activated FAK at 15 min (Fig. 5A), and ERK activation was observed between 15 and 30 min (Fig. 5B). Pretreatment with an integrin αvβ3 mAb significantly suppressed activation of both FAK and ERK, indicating that TSP4 induces FAK and ERK signaling in LECs through the integrin αvβ3 receptor (Fig. 5C, D). Furthermore, application of a FAK inhibitor (FAKi) markedly reduced TSP4-induced ERK activation, suggesting that FAK acts upstream of ERK in this signaling pathway (Fig. 5E). Finally, treatment with either a FAKi or an ERK inhibitor (ERKi) significantly suppressed TSP4-induced LEC recruitment (Fig. 5F). In conclusion, these results demonstrated that TSP4 secreted by BLCA cells promotes LEC migration into the TME via activation of the integrin αvβ3/FAK/ERK signaling pathway.

### TSP4 cooperates with VEGF-C to promote lymphangiogenesis

**Figure 6:**
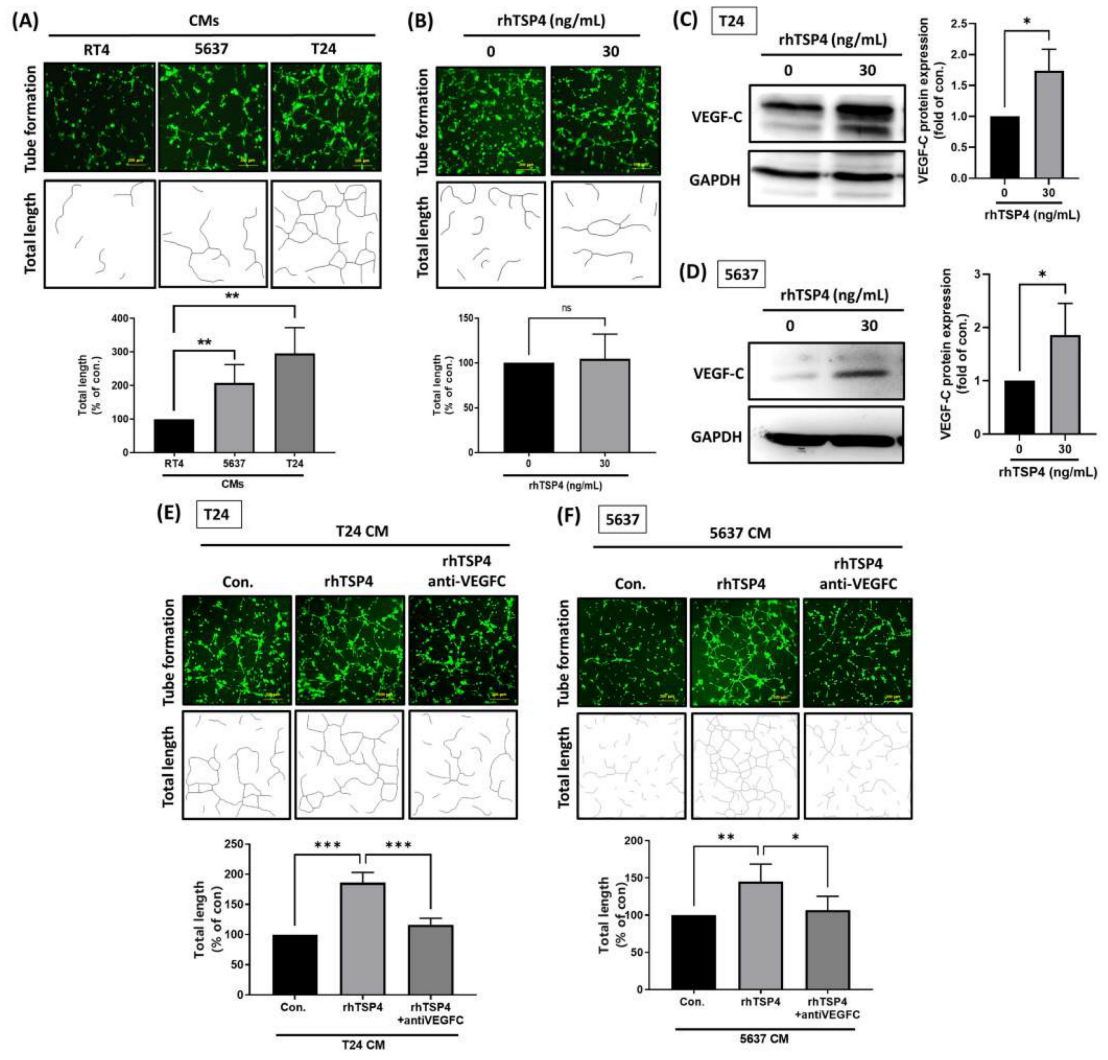
** TSP4 cooperates with VEGF-C to promote lymphangiogenesis.** (A) Representative tube formation images of LEC incubated with CMs from RT4, 5637, and T24 BLCA cells (scale bars, 200 µm). (B) After pretreatment of LECs with or without rhTSP4 (30 ng/mL), a tube formation assay was performed to analyze the effect of TSP4 on lymphangiogenesis (scale bars, 200 µm). Total lengths were quantified by ImageJ software. (C, D) Western blot analysis of VEGF-C protein expression in T24 and 5637 cells treated with or without rhTSP4 (30 ng/mL). (E, F) T24 and 5637 cells were treated with rhTSP4 (30 ng/mL), and the resulting CMs were collected after 24 h. These CM samples were then pre-incubated with a VEGF-C monoclonal antibody (0.5 μg/mL) for 30 min prior to the tube formation assay (scale bars, 200 µm). Total tube lengths were quantified. Data are presented as mean ± SD values. * *p* < 0.05, ** *p* < 0.01, *** *p* < 0.001, compared to the control group.

VEGF-C plays a crucial role in LNM of multiple malignancies 26. However, little is known about whether TSP4 is involved in VEGF-C-regulated lymphangiogenesis. Therefore, we first assessed the ability of CMs from BLCA to induce lymphatic tube formation by LECs using a lymphatic tube-formation assay. Results showed that CM from T24 cells induced the highest level of lymphatic tube formation, followed by CMs from 5637 and RT4 cells (Fig. 6A). However, treatment with rhTSP4 did not affect LEC tube formation (Fig. 6B), suggesting that TSP4 does not directly promote this process.

The Western blot analysis revealed that TSP4 upregulated VEGF-C protein expression in BLCA cells (Fig. 6C, D). Furthermore, T24 and 5637 cells were induced with rhTSP4, and their CMs were collected. Pretreatment with a VEGF-C mAb effectively attenuated the lymphangiogenesis induced by rhTSP4 CMs from T24 or 5637 cells (Fig. 6E, F), suggesting that TSP4 promotes lymphangiogenesis in the TME in concert with VEGF-C.

### The TSP4/VEGF-C axis promotes lymphatic metastasis in BLCA

**Figure 7:**
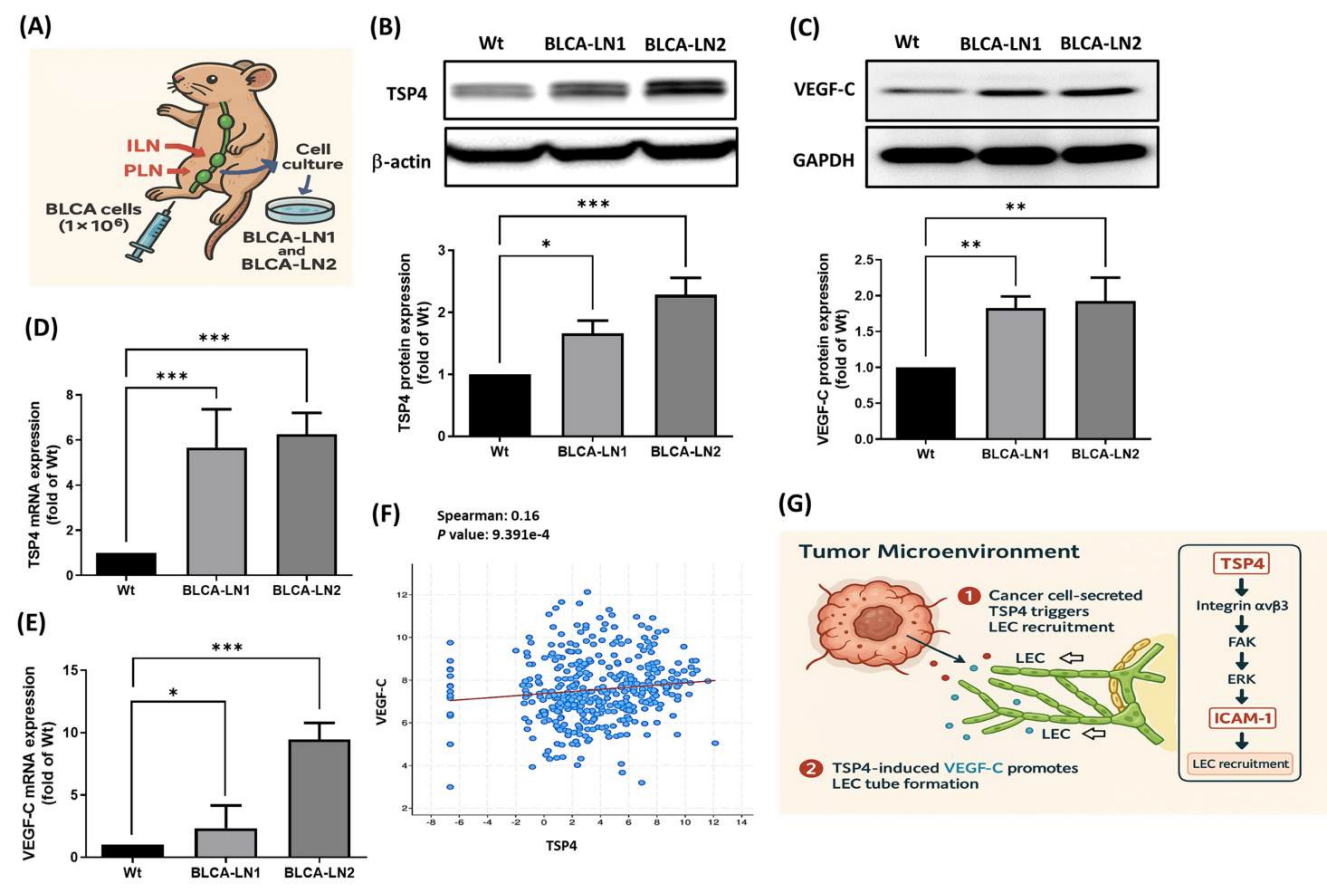
** TSP4 and VEGF-C cooperation promotes lymphatic metastasis in BLCA.** (A) Flow chart of establishing BLCA-LN1 and BLCA-LN2 cells using a PLN animal model. (B-E) Protein and mRNA expression levels of TSP4 and VEGF-C were analyzed by Western blotting and RT-qPCR, respectively. (F) Correlation analysis of VEGF-C and TSP4 expressions was performed using TCGA-BLCA database. (G) Schematic model illustrating that BLCA-derived TSP4 recruits LECs into the TME by upregulating ICAM-1 via the integrin αvβ3/FAK/ERK signaling pathway (1), and further induces VEGF-C expression in BLCA cells, which promotes tube formation of recruited LECs, ultimately leading to lymphangiogenesis within the BLCA TME (2). Data are presented as mean ± SD values. * *p* < 0.05, ** *p* < 0.01, *** *p* < 0.001, compared to the control group.

To further investigate whether TSP4 cooperates with VEGF-C to regulate lymphatic metastasis in BLCA, a PLN metastasis animal model was established to isolate lymph node-tropic BLCA cell lines (BLCA-LN1 and BLCA-LN2). BLCA cells were injected into the footpads of nude mice, and after 6 weeks, metastasis to both PLNs and inguinal lymph nodes (ILNs) was observed. ILN tissues were harvested to isolate and expand the metastatic cancer cells. To stabilize the lymphatic metastatic phenotype, isolated cells were reinjected for a second round of PLN metastasis modeling. After the second round, metastatic cells from two individual ILNs were isolated and designated BLCA-LN1 and BLCA-LN2 (Fig. 7A). Western blotting and an RT-qPCR confirmed that TSP4 and VEGF-C were overexpressed at both the protein and mRNA levels in BLCA-LN1 and BLCA-LN2 cells compared to wild-type BLCA cells (Fig. 7B-E). Furthermore, TCGA-BLCA database analysis verified a positive correlation between TSP4 and VEGF-C expression levels in human BLCA tissues (Fig. 7F).

In summary, BLCA-derived TSP4 recruits LECs into the TME by upregulating ICAM-1 expression through the integrin αvβ3/FAK/ERK signaling pathway. Subsequently, BLCA-expressed VEGF-C promotes lymphatic tube formation in recruited LECs, ultimately contributing to lymphangiogenesis in the BLCA TME (Fig. 7G).

## Discussion

The pathological stage of BLCA is closely associated with the presence of LNM. According to the 2002 TNM classification system, patients with positive lymph nodes are categorized into three stages—N1, N2, and N3—based on the number and size of metastatic lymph nodes 27. Lymphatic metastasis to regional lymph nodes is closely associated with the formation of new lymphatic vessels, a critical mechanism underlying tumor dissemination 28. This process involves the active recruitment of LECs into the TME, primarily mediated by lymphangiogenic factors secreted by cancer cells. These factors stimulate LEC migration toward the tumor, where they subsequently organize into functional lymphatic structures around or within the tumor mass, thereby facilitating the lymphatic spread of malignant cells 29. TSP4 traditionally regarded as an angiostatic factor, participates in diverse biological processes including tissue remodeling, inflammation, and vascular regulation 30-32. Notably, recent studies reported elevated TSP4 expression across various cancer types, where it contributes to tumor progression and metastasis 24. In our previous study, we analyzed TSP4 expression in BLCA patient tissues across different tumor grades (Grade 2-4) and pathological stages (T1-T4), and observed a progressive increase in TSP4 expression with higher grades and more advanced stages. Furthermore, elevated TSP4 levels were associated with poorer overall survival and disease-free survival 20. In this study, we employed three BLCA cell lines representing different clinical stages—RT4 (stage I), 5637 (stage II), and T24 (stage III)—to explore the role of TSP4 in lymphatic metastasis. Our findings demonstrated a positive correlation between TSP4 overexpression and LNM in BLCA, suggesting that TSP4 could possibly serve as a novel therapeutic target for the prevention of lymphatic dissemination in BLCA.

TSP4 is a matricellular protein composed of multiple repeated sequence motifs that enable interactions with ECM components, cell surface receptors, and soluble ligands 33, 34. Through these interactions, TSP4 initiates downstream signaling cascades that regulate diverse physiological processes, including cell adhesion, migration, angiogenesis, and tissue remodeling 20, 32, 35. Among its known binding partners, integrins—particularly integrins α2, αMβ2 and αvβ3—were identified as specific receptors for TSP4 36, 37. These integrins mediate TSP4-induced responses in various pathological contexts. For instance, TSP4-αMβ2 binding was implicated in promoting proinflammatory and proatherogenic stimuli in vascular endothelial cells, contributing to lesion development in cardiovascular disease models 38. In the context of cancer biology, integrin αvβ3 is of particular interest due to its high expression in both tumor and stromal compartments, where it facilitates processes such as angiogenesis, invasion, and metastasis 39-41. Moreover, ICAM-1, a member of the immunoglobulin super-family, mediates cell-cell adhesion and links to the actin cytoskeleton via α-actinin or ezrin, facilitating lamellipodium formation 42-44. It further contributes to the establishment of cell polarity and modulates endothelial cell migration through mechanisms involving endothelial nitric-oxide synthase activation and actin cytoskeleton remodeling 21. In this study, we identified ICAM-1 as a critical mediator of TSP4-induced LEC motility. Mechanistically, TSP4 activates the integrin αvβ3/FAK/ERK signaling axis, leading to enhanced LEC migration, which may contribute to lymphangiogenesis and lymphatic metastasis in the BLCA TME. These findings not only reinforce the role of TSP4 as a pro-metastatic factor but also identify ICAM-1 and integrin αvβ3 as potential therapeutic targets to disrupt TSP4-mediated lymphatic dissemination in BLCA. Importantly, interventions targeting TSP4 or its interaction with integrin αvβ3 may hold translational potential, providing a strategy to inhibit lymphatic metastasis and potentially improve clinical outcomes for BLCA patients.

VEGF-C, a prominent lymphangiogenic growth factor, potently induces lymphangiogenesis. Inhibition of VEGF-C signaling by neutralizing antibodies was shown to suppress lymphatic metastasis 12, 13. An mAb targeting VEGF-C (VGX-100) has reportedly undergone phase I clinical trials for various metastatic tumors. In combination with Avastin (bevacizumab), VGX-100 exhibited broad-spectrum inhibitory effects on both angiogenesis and lymphangiogenesis (ClinicalTrials.gov NCT01514123). Moreover, clinical trials focusing on lymphangiogenesis signaling pathways are currently in progress. Neutralizing antibodies against the VEGF-C and VEGF-D signaling pathways have demonstrated effective suppression of tumor lymphatic metastasis in both preclinical models and early-phase clinical trials 45. Numerous studies have established a direct correlation between LNM and aberrant expressions of specific biomarkers 46, 47. Consequently, a comprehensive understanding of the regulatory mechanisms underlying LNM, alongside the early identification and therapeutic intervention in high-risk patients via these biomarkers, represents a promising strategy to enhance survival outcomes. Nevertheless, the exact relationship between the TSP4/VEGF-C axis and tumor lymphangiogenesis within BLCA remains ambiguous. In the present study, our findings indicate that TSP4 does not significantly alter the capacity of LECs to form lymphatic tubes, but it does induce VEGF-C expression. This suggests that TSP4 can cooperate with VEGF-C to foster lymphangiogenesis in the TME.

## Conclusions

In conclusion, this study demonstrates the role of TSP4 in promoting lymphangiogenesis in BLCA. We further identified the integrin αvβ3/FAK/ERK/ICAM-1 signaling pathway as a key mediator that enhances the migration and invasion of LECs into the TME. Additionally, TSP4 upregulates VEGF-C expression in BLCA cells, which in turn promotes lymphatic tube formation in recruited LECs, collectively contributing to lymphangiogenesis and lymphatic metastasis.
